# High-throughput phenotyping of nematode cysts

**DOI:** 10.3389/fpls.2022.965254

**Published:** 2022-09-14

**Authors:** Long Chen, Matthias Daub, Hans-Georg Luigs, Marcus Jansen, Martin Strauch, Dorit Merhof

**Affiliations:** ^1^Institute of Imaging and Computer Vision (LfB), RWTH Aachen University, Aachen, Germany; ^2^Federal Research Center for Cultivated Plants, Julius Kühn Institute (JKI), Elsdorf, Germany; ^3^LemnaTec GmbH, Aachen, Germany

**Keywords:** phenotyping, nematode cyst, *Heterodera schachtii*, nematode infestation, sugar beet, CNN

## Abstract

The beet cyst nematode *Heterodera schachtii* is a plant pest responsible for crop loss on a global scale. Here, we introduce a high-throughput system based on computer vision that allows quantifying beet cyst nematode infestation and measuring phenotypic traits of cysts. After recording microscopic images of soil sample extracts in a standardized setting, an instance segmentation algorithm serves to detect nematode cysts in these images. In an evaluation using both ground truth samples with known cyst numbers and manually annotated images, the computer vision approach produced accurate nematode cyst counts, as well as accurate cyst segmentations. Based on such segmentations, cyst features could be computed that served to reveal phenotypical differences between nematode populations in different soils and in populations observed before and after the sugar beet planting period. The computer vision approach enables not only fast and precise cyst counting, but also phenotyping of cyst features under different conditions, providing the basis for high-throughput applications in agriculture and plant breeding research. Source code and annotated image data sets are freely available for scientific use.

## 1. Introduction

Many nematode species, such as the beet cyst nematode (BCN) *Heterodera schachtii*, are parasitic on plants and responsible for losses in crop yield that amount to annual financial losses of more than 150 billion USD (Singh et al., 2015). Screening of soil samples for assessing nematode infestation qualitatively and quantitatively is a preventive measure and an integral part of pest management strategies in agriculture. It is also routinely performed by state institutions for import-export inspections of plants.

Cyst nematodes persist in the soil over many years as eggs inside a cyst, which is a protective shell formed by the remains of the former female body. Once a host plant germinates, juveniles are stimulated to hatch from the eggs and to leave the cyst. Juveniles move through the soil matrix, penetrate the roots and induce a so-called syncytium in the root tissue, causing damage to the plant. A female nematode can produce several hundred eggs. Once the eggs are developed, the female nematode dies and its body wall hardens, forming a brown, sclerotized cyst.

The ability to quantify nematode infestation is a prerequisite for control measures targeted at nematodes, as well as for the development of nematode-resistant plant breeding lines. For example, nematode population density may be estimated by cyst counting: The cysts need to be hand-picked from soil samples that still contain organic debris, where the amount of debris depends on the particular sample extraction method (Hallmann et al., 2020). Manual counting is a time-consuming task, and counting accuracy is affected by subjective decisions, the ability to keep up concentration and the experience of the human counter in separating cysts from other particles. Hence, only automated counting is suitable for high-throughput applications.

While the number of nematodes is an important quantitative measure, phenotypical features, such as size, shape and color of the cysts, are also relevant: It could be shown that increased cyst size is an indicator of adaptation of a potato cyst nematode population to plant resistance (Fournet et al., 2016), suggesting that the cyst phenotype is relevant for resistance applications. While other data modalities, e.g., genetic data, may also be applied to estimating nematode infestation (Bogale et al., 2020), only image data can be used to characterize the phenotype of a cyst.

**Figure 1:**
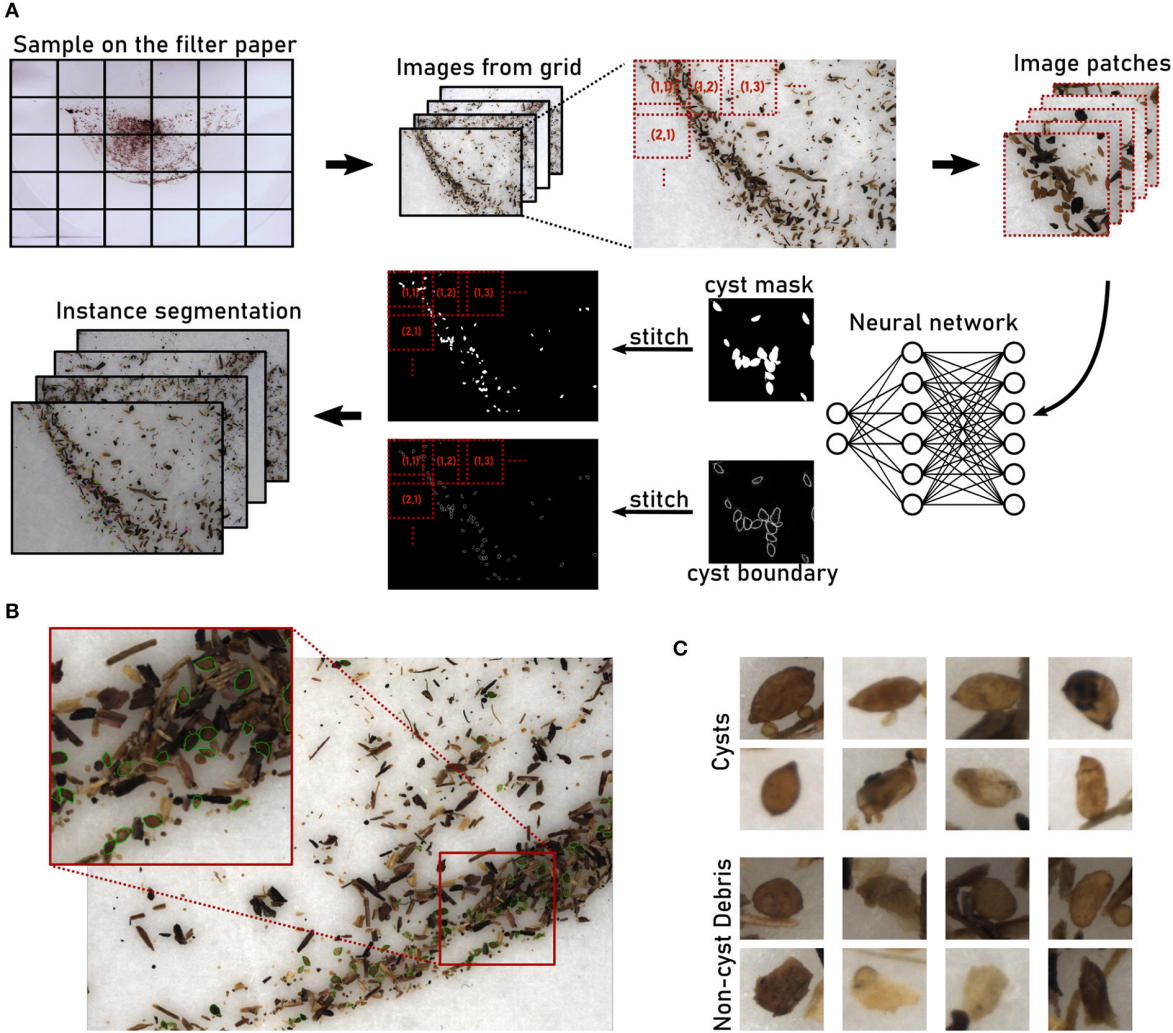
Computer vision pipeline for instance segmentation of nematode cysts. **(A)** A high-resolution image is partitioned into tiles that are processed separately. For each image tile, patches are processed by the neural network to generate cyst and boundary masks. Stitching the tile-level masks together results in whole-image masks that are further processed to obtain the final instance segmentation (Methods). **(B)** Microscopical image of a soil sample extract containing nematode cysts (annotated in green) and organic debris particles. **(C)** Examples for nematode cysts and debris particles that resemble the cysts.

Here, we introduce an automated system based on computer vision that serves as the basis for extracting quantitative measures of nematode infestation from soil samples (Figure 1A). An optical microscopy readout followed by instance segmentation to detect individual cysts surrounded by organic debris particles (Figure 1B) enables fast processing in a high-throughput manner, while also providing access to phenotypical features. The system relies on a supervised learning model trained to detect cysts of the nematode *Heterodera schachtii* that is primarily parasitic on sugar beets. With additional training data, the system could be generalized to other nematode species as well.

We have previously performed nematode cyst instance segmentation on images that are comparable to the image data from this paper, being recorded with a preliminary version of our image recording hardware: Chen et al. (2019) have proposed a method for cyst instance segmentation that relies on instance proposals followed by instance classification with a support vector machine (SVM). The method was designed for small data sets, where deep learning networks do not perform well due to a lack of training data. For the large annotated data sets from this paper, we employ deep learning strategies instead.

Here, we focus on the detection of intact nematode cysts as they occur in unprocessed soil sample extracts. Once the cysts are broken up, e.g., through crushing, the eggs or juvenile nematodes contained therein will be released. Nematode egg detection has been performed by Akintayo et al. (2018), Kalwa et al. (2019), and Chen et al. (2020) have considered juvenile detection. We plan to include these additional instance segmentation scenarios into a future version of the high-throughput phenotyping system.

The nematode egg detection scenario from Akintayo et al. (2018) is similar to our work in that relatively rare target objects need to be detected amidst a large number of cluttered distractor objects. The authors also employ a deep learning strategy for this detection task. While they consider processed samples to which fuchsin acid staining has been applied and that contain crushed cysts, our approach enables high-throughput applications without the need for physical treatment or for staining that can interfere with the phenotypical features.

In this work, we introduce a computer vision based system for high-throughput phenotyping of nematode cysts in extracts from soil samples. This is a challenging scenario with target cysts immersed in a large number of distractors, such as soil particles and plant seeds. For the purpose of model training and evaluation, we created three image data sets with annotated segmentation masks or counts of cysts: *Cyst-Segmentation, Cyst-Count* and *Cyst-Count-Artificial* (Section 2). An extensive evaluation shows that our system is able to detect and segment nematode cysts with high accuracy (Sections 3.1–3.3). In addition, we present a use case for cyst phenotyping, demonstrating how cyst populations can be characterized morphologically (Section 3.4).

## 2. Materials and methods

### 2.1. Sample collection and preparation

Soil samples were taken from fields with a known infestation history of the beet cyst nematode *Heterodera schachtii*. Samples came from different field plots (1.8 × 4.5 m) distributed in a traditional sugar beet growing area in the Rhineland (Western Germany) and from an experimental field at the Julius Kühn-Institute field station in Elsdorf, also situated in the same growing region (50°5541.27N; 6°3375.27 E). The samples were compiled by mixing and homogenizing soils from 10 different plots with a comparable population density, either from the top soil (0–30 cm) or the sub soil (31–60 cm). Using the semi-automatic soil sampler system NH120 (Nietfeld, Badbergen, Germany), 12 evenly distributed soil cores were taken from each plot.

Soil extracts for image acquisition were prepared using a sieve combination (2 mm and 100 μm). Subsequently, cysts were extracted from sieve residues by the centrifugation flotation technique (Viaene et al., 2020) using a high capacity centrifuge (Avanti J-HC, Beckman Coulter, Brea USA). The centrifugation speed was set to 3,000 g. Sieve residues were mixed into a MgSO_4_ solution (1.26 g/ml) to facilitate buoyancy of the organic fraction. For each sample, 100 or 300 g of field fresh soil were processed, resulting in a soil extract containing a number of cysts ranging from zero to several hundreds (Table 1). After pouring the centrifugate into a funnel, cysts along with the remaining organic fraction were collected on a white filter paper (185 mm, MN 616 Macherey Nagel, Germany). A second filter paper put underneath absorbed excess water to avoid reflectance effects on the sample surface caused by a light source above the sample.

**Table 1:** Samples used for cyst count validation.

**Dataset**	**Sample**	**Replicates**	**Images**	**Weight**	**Cysts**	**Description**
Cyst-Count	Top-Low-A debris/clean	6	6x(30 + 12)	300 g	635	top soil, low cyst density
	Top-Low-B debris/clean	6	6x(30 + 12)	300 g	1,122	
	Top-High-B debris/clean	6	6x(30 + 12)	300 g	1,616	
	Sub-Low-B debris/clean	6	6x(30 + 12)	300 g	505	
	Sub-High-B debris/clean	6	6x(30 + 12)	300 g	900	
Cyst-Count- Artificial	Cyst30-100	6	6 × 30	100 g	6 ×30	30 cysts with debris
	Cyst30-300	6	6 ×30	300 g	6 ×30	
	Cyst60-300	6	6 ×30	300 g	6 ×60	
	Cyst120-300	6	6 ×30	300 g	6 ×120	
	Cyst240-300	6	6 ×30	300 g	6 ×240	
Debris	Debris-Cyst120-100	6	6 ×30	100 g	0	debris of Cyst120-100
	Debris-Cyst120-300	6	6 ×30	300 g	0	debris of Cyst120-300
	Debris-Cyst240-300	6	6 ×30	300 g	0	debris of Cyst240-300

The *Cyst-Count* data set contains samples from the top soil (0-30 cm) and from the sub soil (31-60 cm) of the sugar beet field (factor *soil_type*). Samples A and B were collected after (in October, 2020) and before (in April, 2021) the sugar beet planting season, respectively (factor *time_point*). In each case, there are 30 images of samples with debris particles and 12 images of manually cleaned samples without debris. A manual cyst count serves as the ground truth. For the *Cyst-Count-Artificial* data, different numbers of hand-picked cysts were added to cyst-free extracts of soil samples. The cyst-free extracts alone served as an empty control referred to as the *Debris* data.

### 2.2. Image recording

Images of the samples were recorded by JKI (Julius Kühn Institute) with a PhenoAIxpert HM prototype (Supplementary Figure S3) provided by LemnaTec GmbH (Aachen, Germany). It consists of an industrial RGB camera (resolution: 41, 12 × 3, 008 pixels) combined with a high-magnification lens system (1 × magnification with 0.25 × lower lens). The system is mounted 35 cm above the object and has a field of view of 35 × 25 mm. Samples are illuminated with horizontally oriented LEDs (4, 000 K) arranged around the sample stage. All components are mounted inside an opaque cabinet that shields them from external light.

In the PhenoAIxpert HM prototype, the sample stage consists of a sample holder that can be moved manually to predefined positions, such that the complete surface of the sample can be imaged in a series of photographs. Image acquisition is triggered manually with a control computer. In the future, the sample stage will be replaced by a motorized X-Y table, which will then automatically move the sample to predefined positions and trigger image acquisition in a synchronized manner.

### 2.3. Data sets and annotation

In order to validate the cyst detection and segmentation performance of the automated system, i.e., image recording followed by the computer vision pipeline, we created three image data sets and associated evaluation scenarios:

- *Cyst-Segmentation*: Segmentation scenario. Cyst boundaries were manually annotated on the images. The data set consists of 229 images with a total of 6, 331 annotated cysts. We randomly split the data into the 80% training set (183 images containing 4, 937 cysts) and the 20% test set (46 images containing 1, 394 cysts). The training set was used for training of all models in this work, and the test set served for evaluation of segmentation accuracy (Section 3.3). Considering that cysts surrounded by debris particles can easily be overlooked, two raters labeled the data independently with the polygon tool in the annotation software IMANNO developed by LfB (publicly available). The two sets of annotations were then manually merged and validated by a nematologist (co-author Matthias Daub).
- *Cyst-Count* (Table 1): Cyst counting scenario. The ground truth cyst count was obtained through manual counting. We considered soil samples from two different soil layers (top soil: 0–30 cm, sub soil: 31–60 cm) with either low or high cyst density. For each soil type (Top-Low, Top-High, Sub-Low and Sub-High), we conducted soil sampling before and after the sugar beet growing season, 6 samples each time. A total of 48 samples (4 types ×2 timepoints ×6 samples) were collected, processed and recorded with 30 images per sample (the “debris” sample). For each sample, we further manually separated the cysts from debris particles to obtain the “clean” samples (12 images per sample). Cyst separation and counting were performed simultaneously by the professional laboratory staff from JKI.
- *Cyst-Count-Artificial* (Table 1): Cyst counting scenario. We artificially created samples with exact cyst numbers by adding hand-picked cysts to cyst-free soil extracts. We controlled the cyst count (30, 60, 120, and 240 cysts) and the amount of soil extracts (from 100 to 300 g soil sample), generating 6 samples for each combination.

### 2.4. Computer vision pipeline for cyst segmentation

We employed a deep learning model with a convolutional neural network (CNN) in a semantic segmentation setting, performing pixel-level classification of “cyst vs. non-cyst,” as well as “boundary vs. non-background.”As the images enclose a large field of view, they had to be processed patch-wise in a split&stitch manner due to GPU memory limitations. A semantic segmentation map is simpler and more efficient to stitch than the instance label map generated by detection-based approaches such as Mask-RCNN (He et al., 2017) and YOLO (Bochkovskiy et al., 2020). After stitching together the patch-level maps to obtain the image-level semantic segmentation maps for “cyst” and “boundary,” we combined them to compute the final image-level instance segmentation map. For a flowchart of the computer vision pipeline, see Figure 1A.

***Network architecture***. The network takes three-channel RGB images as input. Raw pixel intensities are linearly scaled, such that every image has zero mean and a standard deviation of one. As the backbone network, we chose a U-Net (Ronneberger et al., 2015). In our U-Net implementation, we follow the typical U-Net architecture, but each convolutional layer is preceded by a batch normalization layer before activation.

We also experimented with two ResNet (He et al., 2016) variants (ResNet 50, ResNet 101). ResNet has been proposed for and trained in an image classification setting. Here, we designed a segmentation model based on the original ResNet architecture: First, the fully connected layer and the global average pooling layer are removed, making the model fully convolutional. Then, we construct the decoding path with feature maps from the last convolutional layer of the conv1, conv2, conv3, conv4, and conv5 block, which have 1/2, 1/4, 1/8, 1/16, and 1/32 of the original image resolution, respectively. The decoding path starts from the feature map with the lowest resolution and aggregates features iteratively by upsampling the lower resolution feature map and concatenating it with the one from the next resolution level:


$$\begin{array}{ll} f_{i}^{\prime } & =U_{i}\left(f_{i}^{*}\right),\;i=1,2,3,4,5\\ f_{i} & =C\left(f_{i}^{*},f_{i+1}^{\prime }\right),\;i=1,2,3,4\\ f_{out} & =U_{out}\left(f_{1}\right) \end{array}$$

where $f_{i}^{*}$ is the feature map extracted from the i-*th* conv block of ResNet. $U_{i}$ and $U_{out}$ upscale the feature map *via* a transposed convolution layer with a 2 × 2 kernel and a 2 × 2 stride. C denotes a channel-wise concatenation of feature maps. For each concatenation, we keep the feature maps, $f_{i}^{*}$ and $f_{i+1}^{\prime }$, balanced with the same channel number. Therefore, the filter number of the transposed convolution layer *U*_*i*_ is determined by the channel number of feature map $f_{i-1}^{*}$.

At the output end of the network, we pass the feature map *f*_*out*_ to two independent convolutional layers, which are responsible for cyst and boundary recognition, respectively:


$$\begin{array}{ll} S_{cyst} & =\sigma \left(conv_{cyst}\left(f_{out}\right)\right),\\ S_{boundary} & =\sigma \left(conv_{boundary}\left(f_{out}\right)\right) \end{array}$$

Both convolutional layers, *conv*_*cyst*_ and *conv*_*boundary*_, use a single 1 × 1 convolutional kernel, and the outputs are activated with a sigmoid function σ(*x*) = 1/(1+exp(−*x*)).

***Model training***. We trained the model on the images from the training set of the *Cyst-Segmentation* data and then applied it to the test set, as well as to the other data sets from the counting scenarios (Table 1) that served as additional test sets.

For model training, we split the original images into 512 × 512 patches. Patches with less than 500 cyst pixels were ignored. We randomly selected 10% of the extracted patches for validation. The rest training patches were augmented: In order to not interfere with phenotypical features, such shape and color, only random rotation, random Gaussian blur (σ = 3) and random gamma transformation (γ∈[0.5, 2]) were performed.

The model was trained with the standard binary cross-entropy loss *ce*(*p*1, *p*2) = −*p*_2_log(*p*_1_)−(1−*p*_2_)log(1−*p*_1_), both for cyst prediction and for boundary prediction:


$$Loss=CE(S_{cyst},\;C)+CE(S_{boundary},\;B)$$

The function *CE* computes the mean value of *ce* applied element-wise to every pixel. **C** and **B** are the binary maps for “cyst” and “boundary,” respectively. To obtain the boundary map **B**, we performed a morphological dilation and erosion on the label map, using a disk-shaped structuring element of radius 2. The difference between the dilated and eroded label map was taken as the boundary training map.

We trained the models for about 550 k iterations with the batch size of 4, using the RMSprop optimizer (Hinton et al., 2014) with a gradient decay of 0.9. The learning rate was 0.0001 initially and decreased exponentially to 90% of its previous value every 10, 000 steps. During training, we saved the best model based on the validation metric AJI (Kumar et al., 2017). Model building and training was performed with Tensorflow (version 2.6).

***Split&stitch processing***. We employed a split&stitch strategy in order to be able to process images encompassing a large field of view. After splitting an image into patches, we computed score maps for “cyst” and “boundary” for each patch using the trained CNN model. Then, score maps at the patch level were stitched together to obtain the score map at the image level.

1. *Splitting and patch processing*: An image was split into overlapping patches of size 512 × 512 (256 pixels overlapped). Each patch was processed separately by the trained CNN model, obtaining a cyst score map and a boundary score map.
2. *Stitching*: Patch predictions were stitched back to the original positions in the image. In the region of overlap, we averaged over the cyst scores from different patches, whereas for the boundary score, we took the maximum. This was motivated by the fact that the boundary is the more vulnerable structure and that successful separation of adjacent cysts relies on a continuous boundary segment between them. For stitching, we also removed the outermost 64 pixels that contain more erroneous predictions due to partial visibility of cysts.

***Instance segmentation***. Finally, we obtained an instance segmentation from the image-level semantic segmentation.

1. *Instance separation*: We employed a threshold of 0.5 to distinguish cyst pixels from background pixels in the cyst map. In the same way, boundary pixels were obtained from the score map. By excluding cyst pixels that were also boundary pixels, cyst instances could be separated spatially. Instances on the binary, thresholded map were then uniquely labeled by connected components labeling (8-neighbor connection).
2. Post-processing: Cyst pixels not assigned to any cyst instance were merged to the closest one. Furthermore, noisy predictions were removed based on a safe minimal size (500 pixels).

### 2.5. Evaluation metrics

For the fully annotated data set *Cyst-Segmentation*, we matched each predicted object to the ground truth object with the largest intersection over union (IoU). Using a certain IoU threshold, a match can be considered as a success (threshold exceeded) or a fail. The successful matches define the True Positives (TP), while predicted objects that have no successful match are False Positives (FP), and ground truth objects without successful matches are False Negatives (FN). Accumulating these values through images of the whole data set, a precision metric (P) can be defined as:


$$\begin{array}{l} P_{IoU}=\frac{TP_{IoU}}{TP_{IoU}+FP_{IoU}+FN_{IoU}}. \end{array}$$

By sweeping over a range of IoU thresholds, an average precision under different levels of matching rigor can be computed as an overall metric, taking into account the effect of segmentation accuracy:


$$\begin{array}{l} AP=\frac{1}{\left|IoU\right|}\underset{\begin{array}{c} IoU \end{array}}{\sum }\frac{TP_{IoU}}{TP_{IoU}+FP_{IoU}+FN_{IoU}}, \end{array}$$

where the threshold values range from 0.5 to 0.95 with a step size of 0.05 in this work. Furthermore, we computed the positive predictive value (PPV) to quantify detection accuracy, and the False Negative Rate (FNR) to measure how many objects were missed:


$$\begin{array}{ll} PPV_{IoU} & =\frac{TP_{IoU}}{TP_{IoU}+FP_{IoU}},\\ FNR_{IoU} & =\frac{FN_{IoU}}{TP_{IoU}+FN_{IoU}}. \end{array}$$

We also report the Average PPV (APPV) and the Average FNR (AFNR) over different IoU matching thresholds. As a further measure for segmentation quality, we used the Aggregated Jaccard Index (AJI) (Kumar et al., 2017). Compared to Jarccard Index, AJI accumulates the intersection and union area between ground truth objects and their best matched prediction objects. Thus, AJI not only measures the accuracy of segmentation, but also indirectly reflects the quality of object separation.

## 3. Results and discussion

### 3.1. Evaluation of cyst counting performance on manually annotated images (*Cyst-Count*)

In the *Cyst-Count* scenario, we compared manually annotated and automatically computed cyst counts for a variety of soil sample types. Samples came from two different soil layers (top soil: 0–30 cm, sub soil: 31–60 cm) and had either low or high cyst density (Section 2.3). For all combinations of these categories (Top-Low, Top-High, Sub-Low and Sub-High), we considered samples with cysts and organic debris particles (“debris”), as well as clean samples (“clean”) where the cysts had been manually separated from the debris particles.

**Figure 2:**
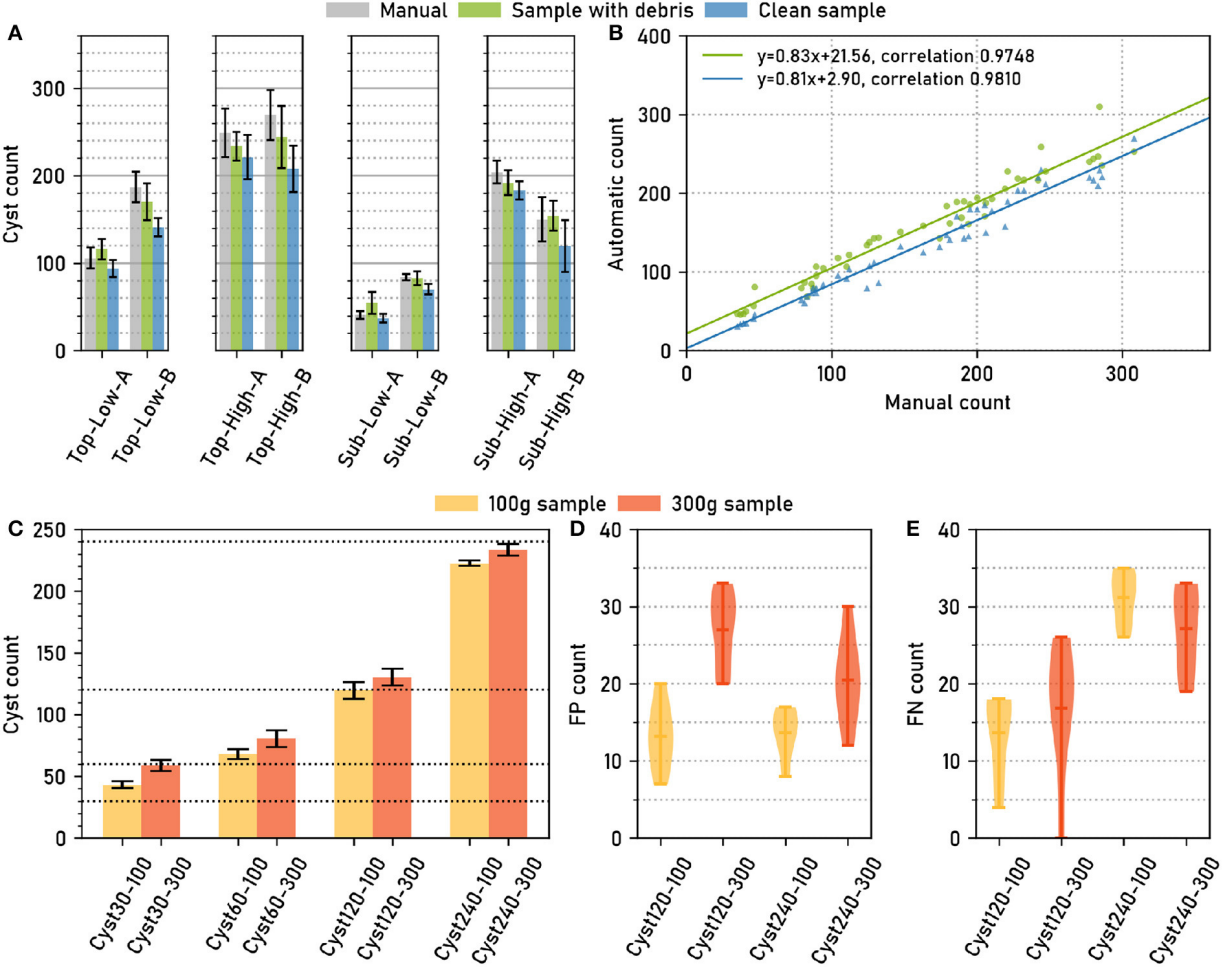
The computer vision pipeline produces accurate cyst counts: Comparison with manual counts. **(A)** Data set *Cyst-Count* (Table 1): Manual and automatic cyst count on samples with debris particles, as well as automatic count on clean samples without debris for different types of soil samples. *N* = 6 samples per bar. **(B)** Data set *Cyst-Count*: Manual count vs. automatic count on samples with debris and on clean samples. *N* = 2 × 4 × 6 samples. **(C)** Data set *Cyst-Count-Artificial*: Automatic count on samples with 30/60/120/240 cysts transferred to cyst-free extracts from 100g and 300g soil samples, respectively. The correct cyst numbers are marked by dotted lines. **(D)** Data set *Debris*: Automatic count on the control samples without cysts, i.e., the number of False Positives (FP). *N* = 12 samples per box plot. Mean FP count (100 g sample): 13.42±3.62. Mean FP count (300g sample): 23.75±6.11. **(E)** Data set *Cyst-Count-Artificial*: Estimated number of False Negatives (#FN). We computed *#FN* = *#P*−*#TP*, where *#P* is the correct cyst count and *#TP* = #cyst_detections - *#FP*, using the FP estimate from the *Debris* data in d). Mean FN count (120 cysts): 15.25±7.03. Mean FN count (240 cysts): 29.16±4.81.

Across all soil sample types, manual cysts counts were similar to automatic cyst counts, both for the “debris” and for the “clean” case (Figure 2A). Pearson correlation coefficients for the correlation between manual and automatic count were high, 0.981 for “clean” and 0.975 for “debris” (Figure 2B), showing that the relative amount of cysts could be estimated reliably with the automatic method.

The samples contained between about 50 and about 300 cysts. Fitting a linear regression model to the manual (x) vs. automatic (y) cyst count results revealed a small y offset of only ≈+3 for the automatic cyst count on the clean samples, while the y offset was ≈+22 for the debris samples (Figure 2B). Hence, also the absolute count predictions were highly accurate on the clean samples, while we observed a slight overestimation of the counts on the debris samples.

### 3.2. Evaluation of cyst counting performance on data with an exact ground truth (*Cyst-Count-Artificial*)

The manually annotated ground truth for *Cyst-Count* may still be affected by False Positive (FP) detections, as debris particles can resemble the cysts (Figure 1C). For the *Cyst-Count-Artificial* data set (Table 1), we could obtain an *exact ground truth* by adding 30/60/120/240 cysts to cyst-free soil sample extracts weighing 100 or 300 g, respectively. The latter also served as empty control samples to estimate the number of FPs.

True and predicted cyst numbers were in good agreement for the samples with higher cyst numbers (Figure 2C), although we observed a two-fold overestimation of the cyst count for the 30 cysts/300 g sample, i.e., the sample with the smallest number of cysts and a large amount of debris particles.

Sample weight had an influence on the count: The automatic count for the 300 g samples *with* cysts was consistently higher (12.33±1.94) than for the 100 g samples with the same number of cysts (Figure 2C). On the empty controls *without* cysts, on average 13.42 ± 3.62 FP cysts were detected for the 100 g samples, and 23.75±6.11 FP cysts for the 300 g samples (Figure 2D). The latter is also consistent with the +21.56 y-offset in Figure 2B that corresponds to an overestimation of the same magnitude for the 300g samples from the *Cyst-Count* data with its image-based manual counts.

The number of FPs was dependent on sample weight, as the higher amount of debris particles in the heavier samples caused more FP detections, but it was not dependent on the number of cysts (Figure 2D). Conversely, the number of False Negatives (FNs) showed no clear dependence on sample weight, but it was dependent on the number of cysts in the sample, with higher cyst numbers leading to more FNs (Figure 2E).

The observed number of FP detections was approximately constant for samples of a given weight, and it was low compared to the typical number of hundreds of cysts in a sample: Only for the artificially small test sample with 30 cysts, the average amount of 23.75 FPs on the 300 g samples did cause a substantial overestimation of the true cyst count. For larger numbers of cysts, the constant offset due to FP detections became less and less relevant, such that true and observed count were approximately equal already for the 120 cysts sample. For the largest sample with 240 cysts, the FN dependence on the number of cysts even led to a slight underestimation of the true cyst count (Figure 2C). Hence, for small cyst numbers close to zero, the error is dominated by the constant FP offset, whereas for large cyst numbers the FP offset is negligible and only the linear increase in FNs is relevant.

In summary, the automatic counting procedure achieved an approximately constant miss rate across a range of cyst numbers, as indicated by the high count correlation in Figure 2B, and a FP rate that depended on the sample weight and that was approximately constant given the weight, as indicated by the empty sample analysis in Figure 2D. For all but very small cyst numbers per sample, the automatic count yielded accurate results.

### 3.3. Evaluation of segmentation accuracy with a manually annotated ground truth (*Cyst-Segmentation*)

We next evaluated the accuracy of the nematode cyst segmentations. For the *Cyst-Segmentation* scenario, the 46 test images with in total (according to the ground truth) 1,394 cysts immersed in debris particles had to be segmented. The manually segmented ground truth was provided by a nematologist, while the automatic segmentation was performed with the computer vision pipeline that relies on the U-Net as a backbone network (Methods). For comparison, we also employed two alternative network architectures, ResNet50 and ResNet101 (Methods).

**Figure 3:**
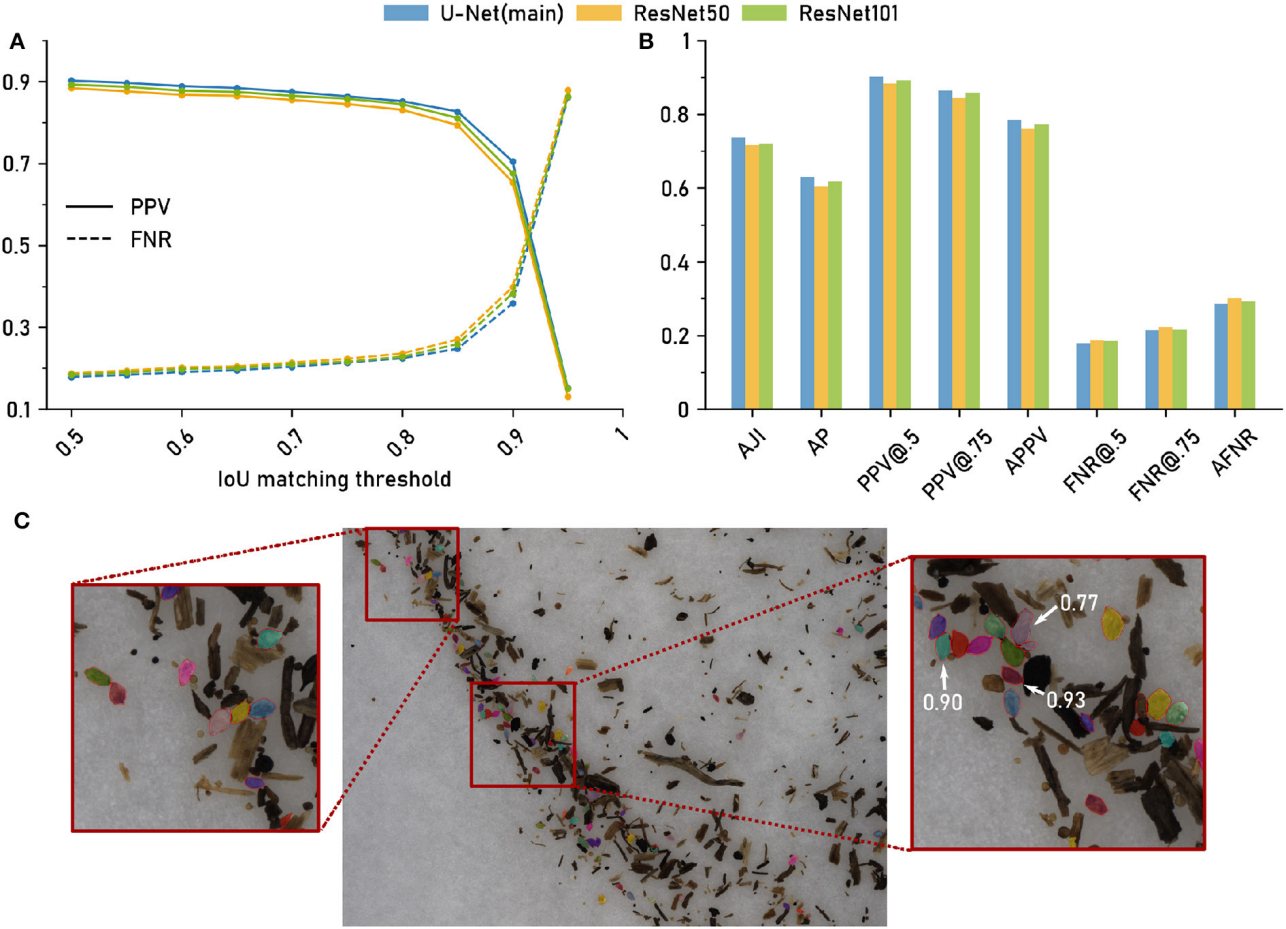
The computer vision pipeline produces accurate cyst segmentations: Comparison with a manually segmented ground truth. Data set: *Cyst-Segmentation*. **(A)** Positive Predictive Value (PPV) and False Negative Rate (FNR) for a range of Intersection over Union (IoU) thresholds. **(B)** Segmentation quality measures (Methods) for the computer vision pipeline with a U-Net backbone and for two architectural variants (ResNet50 and ResNet101 backbone): Aggregated Jaccard Index (AJI), Average Precision (AP), PPV at different IoU thresholds, Average PPV (APPV), FNR at different IoU thresholds, Average FNR (AFNR). **(C)** Qualitative examples for cyst segmentation masks (U-Net). Red lines mark the ground truth boundaries. Selected instance masks are annotated with IoU scores.

Due to the variable number of cysts per image, we chose an evaluation setting based on cumulative scores, i.e., all cysts from all images were pooled. We compute the False Negative Rate (FNR, 1 - recall) and Positive Predictive Value (PPV) at IoU thresholds τ = 0.5, …, 1.0 for each of the three network architectures (Figure 3A).

The same trend could be observed for all three network architectures (Figure 3A): For IoU thresholds up to τ = 0.8, the FNR, that measures the amount of missed cysts, remained constantly at about 0.2, indicating that approximately 20% of the cysts were missed and that 80% could be segmented with such a good IoU. For higher τ, the FNR increased, but was still about 0.5 for τ = 0.9, i.e., half of the cysts could be segmented with a very high IoU of 0.9.

The Positive Predictive Value (PPV, precision), that measures the amount of all true positives among all true and false positives, exhibited the opposite trend, being constantly high above 0.8 up to τ≈0.8 and decreasing for higher τ, crossing the FNR line at τ≈0.9.

While the results were similar for all network architectures, the U-Net had slightly, but consistently, lower FNRs (and slightly higher PPVs) for τ up to about 0.9.

In Figure 3B, we report FNR and PPV for selected IoU thresholds, as well as a number of aggregated metrics, such as Average Precision (AP), that summarize segmentation accuracy across several IoU thresholds (Methods). For all of these evaluation metrics, the three architectural variants performed on a similarly high level.

In summary, we observed only minor performance differences for the alternative networks, indicating that network architecture is not a critical part in the computer vision pipeline as long as a reasonable choice is made. Overall, the computer vision pipeline with the default backbone network yielded accurate instance segmentations with about 80% of the cysts being segmented with a good IoU of about 0.8 or higher. For qualitative results, see the segmentation examples with annotated IoU scores in Figure 3C.

### 3.4. Phenotyping nematode cysts

Finally, we employed the high-throughput-system to provide a use case for cyst phenotyping. We generated segmentation masks for all the cysts that were detected on the *Cyst-Count* data (clean version without debris particles) and then computed two morphological features, cyst size (in pixels) and length-width ratio, to describe the phenotype of a cyst. The *Cyst-Count* data (Table 1) consists of samples taken before and after the sugar beet planting season (factor *time_point* with levels “before season” and “after season”). The samples were extracted either from “top soil” or from “sub soil” (factor *soil_type*). As we observed no interaction between the factors *time_point* and *soil_type* (Supplementary Figure S1), we could pool the samples from both time points for the analysis of the soil type and vice versa.

#### 3.4.1. Phenotypical feature distributions are significantly different between cyst populations on images without debris

**Figure 4:**
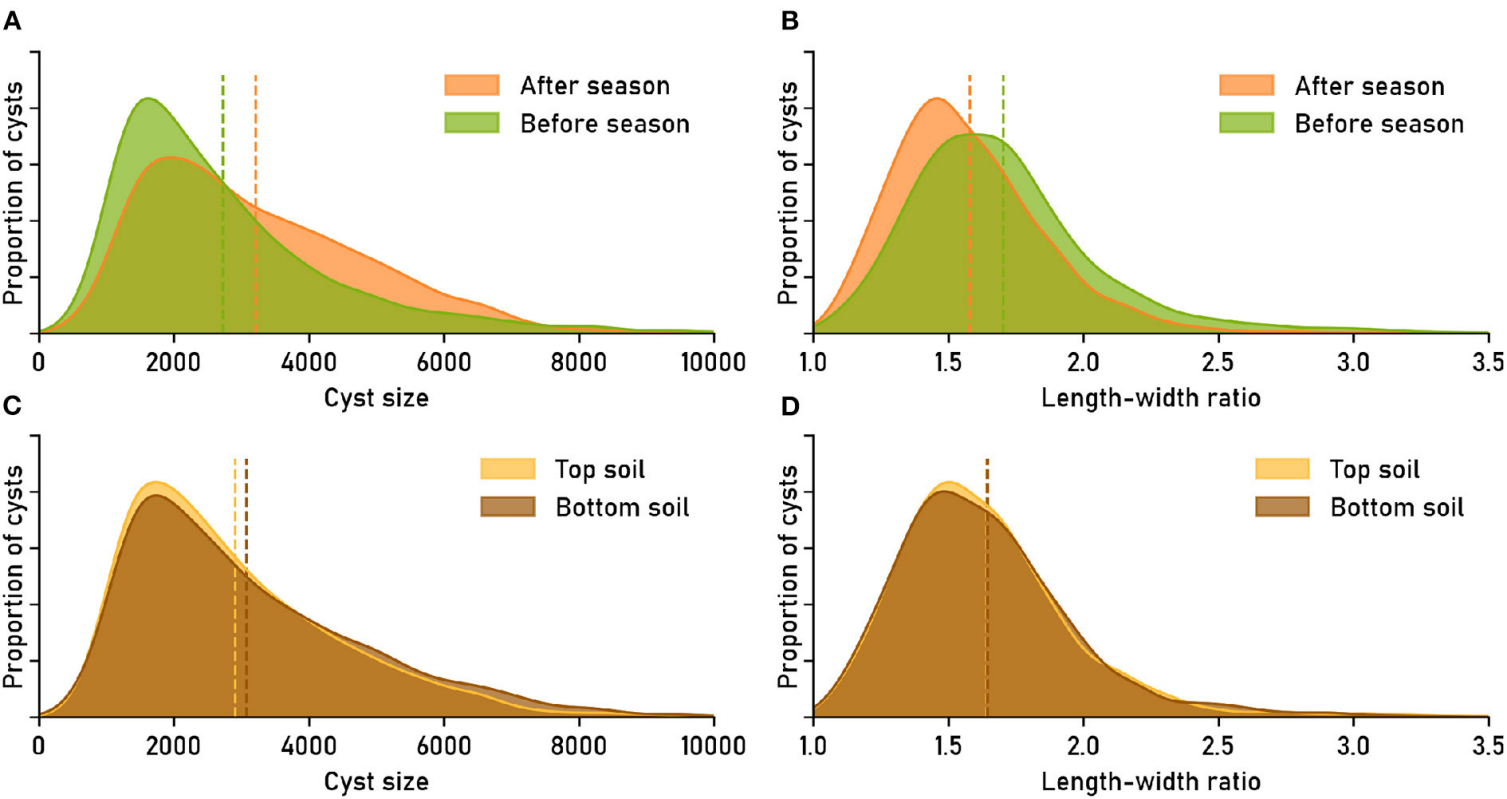
Image-based phenotyping reveals that nematode populations have characteristic phenotypical features. The features “cyst size” (area in pixels) and “length-width ratio” were derived directly from the cyst segmentation masks. Feature distributions show the relative amount of cysts that have a certain feature value. Dashed lines mark the means of the distributions. Data set: *Cyst-Count* (clean version without debris; cp. Supplementary Figure S2 for the version with debris particles). **(A,B)** Nematode cyst populations split by factor *time_point*: sampled “before season” (April) vs. “after season” (October). **(C,D)** Nematode cyst populations split by factor *soil_type*: sampled from “top soil” (0–30 cm) vs. “sub soil” (31–60 cm).

Inspecting the feature distributions, we discovered clearly visible differences between the cyst populations from before and after the planting season (Figures 4A,B). The differences between the populations were significant: Based on the Anderson-Darling k-sample test (Scholz and Stephens, 1987) (R-package kSamples), the null hypothesis that the two populations come from the same distribution could be rejected, in separate tests, both in terms of size (T.AD = 145.6, p = 1.218 × 10^−61^) and in terms of the length-width ratio (T.AD = 154.3, *p* = 2.621 × 10^−65^; Note that we report *p*-values without adjustment for multiple testing).

The feature distribution for cyst size was flatter for the “after season” samples than for the “before season” samples, with less small and more large cysts in the samples that were taken after the planting season (Figure 4A). The average size of cysts after the planting season (≈3, 215 pixels) was about 18% higher than the average size before the season (≈2, 730 pixels). The “after season” distribution for the length-width ratio as a feature was shifted toward smaller ratios compared to the “before season” distribution (Figure 4B), suggesting a generally more circular shape for the cysts extracted later during the year, although the effect was small in absolute numbers (average ratio before: 1.70, after: 1.58).

For the cyst populations extracted from different soil types, the feature distributions appeared more similar (Figures 4C,D). The average cyst size in sub soil (≈3, 074) was slightly larger than in top soil (≈2, 908), while the average length-width ratio was ≈1.64 in both cases. The difference between the feature distributions was only significant for cyst size (Anderson-Darling T.AD = 7.864, *p* = 0.0003168), but not for the length-width ratio (T.AD = −0.8610, *p* = 0.9009).

#### 3.4.2. Phenotypical feature distributions are significantly different between cyst populations on images with debris

The above results on the *Cyst-Count* data (clean version without debris) show how even subtle phenotypical differences become visible in a population level quantitative analysis that is greatly facilitated by an automatic phenotyping system that can annotate large image data sets in a consistent manner. We conducted the population-level study on the cleaned data set in order to explicitly analyze the biological effects, unaffected by FP detections due to debris particles.

We then repeated the same analysis for the *Cyst-Count* data with debris particles (Supplementary Figures S2A–D). While the debris data was noisier, which in part obscured the differences between the distributions, the same trends could be confirmed: There were significant differences with respect to both size (Anderson-Darling T.AD = 13.15, *p* = 2.870 × 10^−6^) and length-width ratio (T.AD = 13.86, *p* = 1.451 × 10^−6^) between the populations from before and after the planting season (Supplementary Figures S2A,B). Regarding soil type (Supplementary Figures S2C,D), a small difference between the size distributions for top and sub soil could still be observed, but it was no longer significant (T.AD = 1.915, *p* = 0.05229), while the difference between the length-width ratio distributions remained non-significant (T.AD = −0.7982, *p* = 0.8554), exactly as for the data without debris particles.

#### 3.4.3. Manually and automatically computed phenotypical feature distributions are not significantly different

Finally, we also verified that the feature distributions based on automatic segmentations were sufficiently similar to the feature distributions based on manual segmentations (Supplementary Figures S2E,F). The analysis was performed on the *Cyst-Segmentation* data (test set) with a ground truth of 1394 manually annotated cyst masks (1, 287 automatically detected cyst masks).

Both average size (manual: ≈2, 223, automatic: ≈2, 226) and average length-width ratio (manual: ≈1.76, automatic: ≈1.73) were similar for the manually and the automatically segmented cysts. The size difference of only about three pixels is negligible for cyst sizes of several thousand pixels, while the 0.03 difference in length-width ratio amounts to 25% of the 0.12 length-width ratio difference observed in Figure 4B for cyst populations from before and after the planting season. Based on Anderson-Darling k-sample tests, the size distributions for the manual and the automatic segmentation were not significantly different (T.AD = −0.5909, *p* = 0.6953), as well as the length-width distributions (T.AD = 1.076, p = 0.1158).

We conclude that the differences between automatic and manual segmentations that exist on the single-cyst level (cp. Figure 3) are of minor importance on the population-level, and that both the size and the length-width ratio distribution can be estimated reliably based on automatic segmentations with the computer vision pipeline.

## 4. Conclusions

We have introduced a high-throughput phenotyping system for nematode cysts in extracts from soil samples (Methods). At the core of the automated system lies a computer vision pipeline that achieves robust and accurate instance segmentation of nematode cysts in cluttered object collections with many remaining debris particles that often resemble the cysts in terms of shape and color (Figures 1B,C).

We have validated the automated system on a large number of images of soil sample extracts from different soils, comparing its results both to manual cyst counts and to manually annotated cyst segmentation masks (Results and Discussion). Manual and automatic cyst counts were highly correlated (Figure 2B; Pearson correlation coefficient >0.97), and about 80% of the automatic cyst segmentations achieved IoU scores of 0.8 or higher (Figure 3A).

The large number of debris particles in the soil sample extracts did cause FP cyst detections. Based on an exact ground truth in the form of artificial soil samples, we could establish that the amount of FPs does not depend on the true number of cysts, but only on the sample weight, i.e., FP detections cause a count offset that is approximately constant (for a given sample weight) and that is only relevant for very small cyst numbers (Figure 2D).

Inaccuracies on a cyst-level had no influence on the population-level phenotypical statistics: The feature distributions for automatically segmented cysts were not significantly different from those of manually segmented cysts (Supplementary Figures S2E,F).

Cyst size and length-width ratio are simple phenotypical features that can be computed from segmentation masks, and we have used them as a proof of concept for automatic characterization of the differences between nematode cyst populations in samples taken before and after the sugar beet planting season, as well as in samples taken from top and from sub soil. Further research is required to determine whether the observed differences between these cyst populations generalize to soils from other locations.

To date, large-scale phenotypical studies on huge, multi-national data sets are infeasible in practice due to intra-rater variability for the manual segmentations and the amount of work involved, and due to differences in experimental setups. With an automated high-throughput phenotyping system, as we have presented it in this work, it becomes possible to conduct large-scale analyses of soil samples from around the world in a reproducible way with a standardized setup and with standardized image analysis routines.

Already cyst size is a morphological feature of high relevance for monitoring nematode resistance (Fournet et al., 2016). Future work will be focused on developing larger phenotypical feature sets based on shape, color and texture of the cysts. The high-throughput system facilitates processing of large data sets from screening studies, enabling phenotypical characterization of nematode cysts under different environmental conditions, in soils from different locations, or in a resistance situation in the face of nematode-resistant plants.

## Data availability statement

The original contributions presented in the study are included in the article/Supplementary materials, further inquiries can be directed to the corresponding author. Source code and evaluation data sets are available online for non-commercial use: https://github.com/looooongChen/PNS-Cyst. The annotation software IMANNO is also publicly available: https://github.com/looooongChen/IMANNO.

## Author contributions

MD, H-GL, MJ, LC, MS, and DM planned research. LC developed machine learning models. MD collected soil samples, designed the sample preparation procedure, and created the annotated evaluation data. H-GL and MJ developed the optical recording setup and recorded images. MS and LC analyzed data and wrote the manuscript with contributions from the co-authors. All authors reviewed the final manuscript.

## Funding

This study received funding from the Germany Ministry of Education and Research (031B0474). The funder was not involved in the study design, collection, analysis, interpretation of data, the writing of this article or the decision to submit it for publication.

## Conflict of interest

Authors LC, MS, and DM are employed by RWTH Aachen University, Aachen, Germany. Author MD is employed by the Federal Research Center for Cultivated Plants (Julius Ku8hn Institute), Elsdorf, Germany. Authors H-GL and MJ are employed by LemnaTec GmbH, Aachen, Germany. The image recording system used in this work, PhenoAIxpert HM, is a prototype that will be rolled out as commercial product by LemnaTec GmbH. The reviewer RH declared a shared affiliation with the author MD to the handling editor at the time of review.

## Publisher's note

All claims expressed in this article are solely those of the authors and do not necessarily represent those of their affiliated organizations, or those of the publisher, the editors and the reviewers. Any product that may be evaluated in this article, or claim that may be made by its manufacturer, is not guaranteed or endorsed by the publisher.
