# Supplementary material for: High-throughput phenotyping of nematode cysts
Source: Front Plant Sci. 2022 Sep 14;13:965254. doi: 10.3389/fpls.2022.965254 (PMC9515587; doi:10.3389/fpls.2022.965254)
Supplement: Supplementary file 1 [file Presentation_1.pdf]

Supplementary Material

1 SUPPLEMENTARY FIGURES

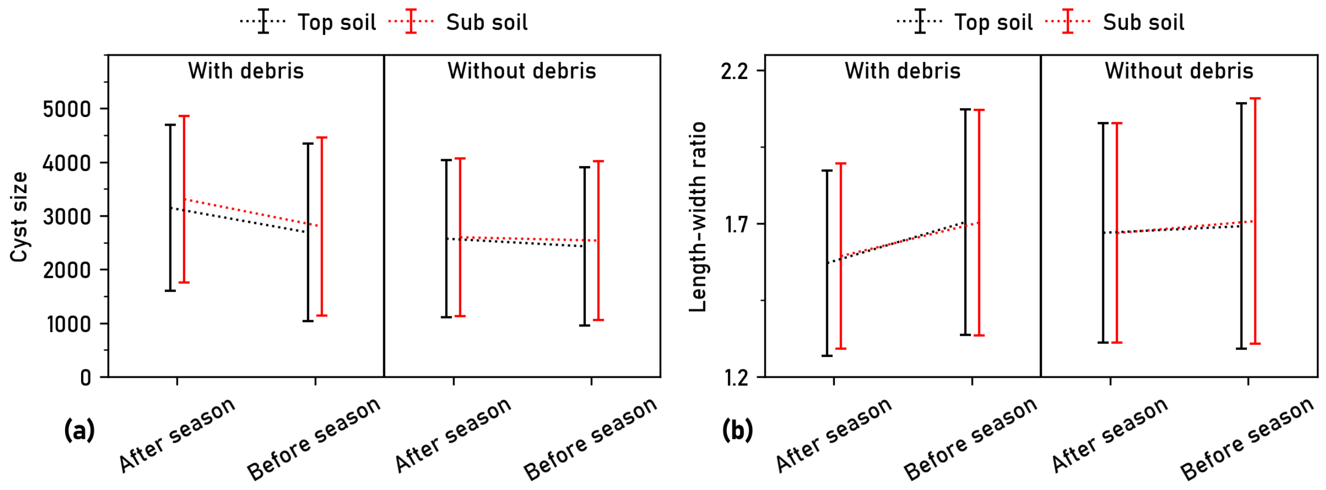

Figure S1. Main effects plots showing that there are no interactions between the factors *soil_type* ("top soil" vs. "sub soil") and *time_point* ("before season" vs. "after season"). ANOVA test (Girden, 1992) reports a p-value of 0.821 for the interaction term. Hence, it is justified to pool the *time_point* levels when comparing "top soil" and "sub soil", and to pool the *soil_type* levels when comparing "before season" and "after season" as done in Figure 4 and Figure S2. Dashed lines connect the means of the respective distributions, and solid lines indicate standard deviations. Data set: *Cyst_count*. Separate plots are shown for the version with and without debris particles. **a)** Feature: cyst size (area in pixels) **b)** Feature: length-width ratio

REFERENCES

Girden, E. R. (1992). *ANOVA: Repeated measures*. 84 (Sage)

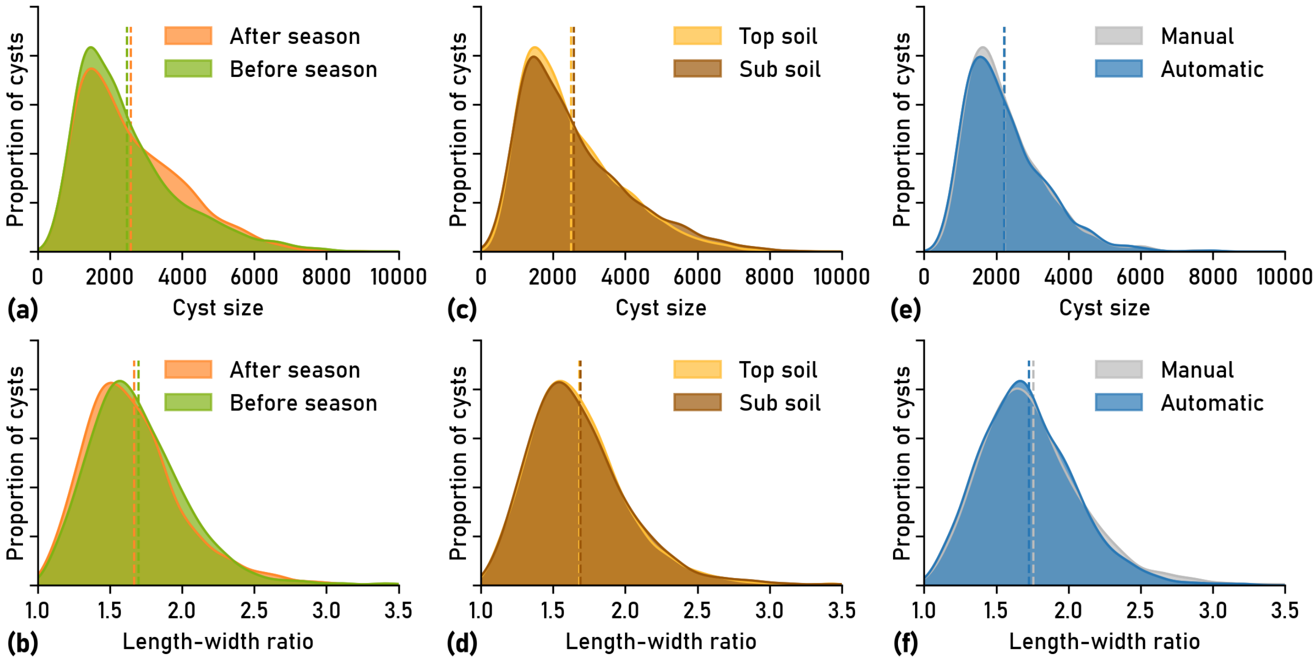

Figure S2. Validation of cyst phenotyping on manually segmented data and on data with debris particles. The phenotypical features ”cyst size” (area in pixels) and ”length-width ratio” were derived directly from the cyst segmentation masks. Feature distributions show the relative amount of cysts that have a certain feature value. Dashed lines mark the means of the distributions. **a) - d)**: Nematode cyst populations split by factor *time_point* (sampled ”before season” vs. ”after season”) and by factor *soil_type* (sampled from ”top soil” vs. ”sub soil”). Data set: *Cyst_count* (version with debris; cp. the clean version without debris particles in Figure 4). **e) - f)**: Feature distributions computed for manually vs. automatically segmented cysts. Data set: *Cyst_segmentation*.

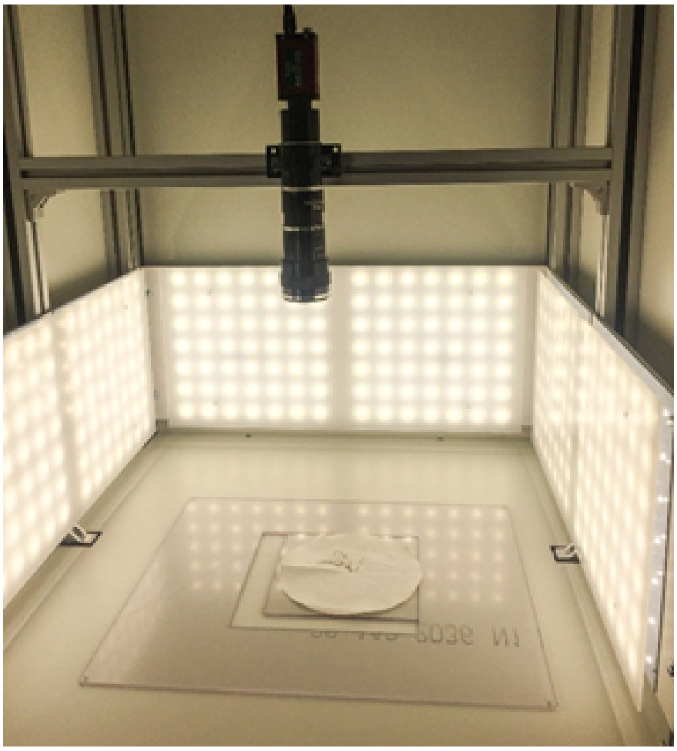

Figure S3. Setup used for recording the microscopical images: LemnaTec PhenoAIxpert HM Prototype (High Magnification).
